# 

**Published:** 1881-11

**Authors:** 


					﻿CONTRIBUTORS:
Dre. H. C. Allen, T. F. Allen, Edward Bayard, J. B. Bell, E. W. Berridge,
Gee. H. Clark, A. C. Cowperthwaite, Ad. Fellger, Wm. J. Guernsey/
W. A. Hawley, John Hall, W. M. James, W. H. Jenny, Ad. Lippe,
C. Lippe, Malcolm MacFarlan, Thos. Moore, C.F. Nichols,
C. Pearson, T. F. Pomeroy, Leila A. Rendell, C, Carle-
ton Smith, P. P. Wells, Wm, P. Wesselhoeft,
David Wilson, (London), T. P. Wilson,
and many others.
NOTICE.
AU articles for publication, books for review, business communications, etc.,
must be sent to Editor, 2109 Chestnut Street, Philadelphia.
Subscribers who have not paid; their Subscrip-
tions will plbase give the matter their
prompt attention.
N. B.-—Authors of accepted papers are furnished with copies of
the number in which their articles appear; application for such
copies to be made when sending papers. Any irregularity in the
receipt of this Journal should be promptly reported to Editor.
A prize of fifty dollars will be awarded to author of the best
essay printed in this Journal for the year 1881. To be adjudged
by three physicians of note.
				

